# A social enterprise model for TB detection and treatment through the private sector in Pakistan

**DOI:** 10.5588/ijtldopen.23.0376

**Published:** 2024-02-01

**Authors:** S. M. A. Zaidi, W. Z. Jamal, U. Ibrahim, S. Khowaja, A. J. Khan, J. Creswell

**Affiliations:** ^1^ WHO Collaborating Centre for Tuberculosis Research and Innovation, Institute for Global Health, University College London, UK;; ^2^ Community Health Solutions, Karachi,; ^3^ Careem, Karachi,; ^4^ Interactive Research & Development, Karachi, Pakistan;; ^5^ Stop TB Partnership, Global Health Campus, Geneva, Switzerland

**Keywords:** social business, social entrepreneurship, sustainability, health systems, public–private mix

## Abstract

**BACKGROUND:**

Existing models to increase TB case notifications from the private sector in Pakistan are financially unsustainable and have achieved modest success due to limited coverage.

**OBJECTIVE:**

To evaluate the impact of a social enterprise model (SEM) intervention on TB case detection in Karachi, Pakistan, and to assess its financial sustainability.

**METHODS:**

Purpose-built centres were established within the private sector that integrated TB screening, diagnostics and treatment and operated 12 hours per day with convenient locations to improve access. TB services were offered free of cost, and revenue generation took place through user fees from other diagnostics. Private providers with a focus on the informal sector were engaged through community workers to generate screening referrals.

**RESULTS:**

Overall 171,488 people were screened and 18,683 cases were notified, including 197 individuals with drug-resistant TB. Annual TB notifications in Karachi increased from 18,105 in 2014 to a maximum of 25,840 (40% increase). The proportion of cases in Karachi notified by the centres grew to 27% in 2020. Commercial revenue reached USD288,065 and enabled operating cost recovery of 15%. Average cost per TB case notified was USD203.

**CONCLUSION:**

The SEM intervention contributed a large proportion of notifications in Karachi and achieved modest cost recovery.

## RÉSUMÉ

**CONTEXTE:**

Les modèles existants visant à augmenter les notifications de cas de TB par le secteur privé au Pakistan ne sont pas viables financièrement et n'ont obtenu qu'un succès modeste en raison d'une couverture limitée.

**OBJECTIF:**

Évaluer l'impact d'un modèle déntreprise sociale (SEM, pour l’anglais « social entreprise model ») sur la détection des cas de TB à Karachi, au Pakistan, et évaluer sa viabilité financière.

**MÉTHODES:**

Des centres spécialisés ont été créés dans le secteur privé. Ils intègrent le dépistage, le diagnostic et le traitement de la TB et fonctionnent 12 heures par jour dans des lieux adaptés afin dén faciliter l'accès. Les services de lutte contre la TB étaient gratuits et les recettes provenaient des frais d'utilisation des autres diagnostics. Des prestataires privés, axés sur le secteur informel, ont été engagés par l'intermédiaire d'agents communautaires pour orienter les patients vers les services de dépistage.

**RÉSULTATS:**

Au total, 171 488 personnes ont été dépistées et 18 683 cas ont été notifiés, dont 197 personnes atteintes de TB résistante aux médicaments. Les notifications annuelles de TB à Karachi sont passées de 18 105 en 2014 à un maximum de 25 840 (augmentation de 40%). La proportion de cas à Karachi notifiés par les centres est passée à 27% en 2020. Les recettes commerciales ont atteint 288 065 USD et ont permis un recouvrement des coûts déxploitation de 15%. Le coût moyen par cas de TB notifié était de 203 USD.

**CONCLUSION:**

L'intervention SEM a contribué à une grande proportion de notifications à Karachi et a permis un modeste recouvrement des coûts.

Pakistan has the fifth highest burden of TB worldwide, with an estimated 573,000 people falling ill each year.^1^ Despite significant progress, over one-third of those estimated to have TB remain undiagnosed.^2^ Increasing case detection and treatment is therefore a key objective for the National TB Programme (NTP).^3^ This can be challenging in countries with complex health systems such as Pakistan, where a majority of health services are delivered through private providers.^4^ While TB services are offered nominally free of charge in the public sector, most people seek care in the private sector due to perceived higher quality and convenience.^5^ Nevertheless, the private sector is fragmented, poorly regulated and inadequately equipped to manage people with TB and providers often do not prioritise TB case-finding.^6^ Care is largely paid through out-of-pocket expenditure and leads to a widening of health inequities.^7^

In recognition of the critical role of private providers, Pakistan’s NTP implemented several public–private mix (PPM) interventions.^8^ While these achieved modest success in increasing notifications, gaps in coverage remained, in particular within the informal sector.^8^ Informal providers were difficult to engage with due to their high numbers, geographic spread and lower volumes. Additionally, PPM initiatives were resource-intensive and needed continuous re-financing for sustainability.^9,10^

Social marketing and social enterprise models (SEMs) have been utilised in other health sectors for increasing access and sustainability, notably in reproductive health.^11–13^ These approaches differ from conventional businesses in that goods or services are sold with a social objective as the primary aim while promoting health access and equity.^14,15^ Social entrepreneurship also differs from conventional charities that offer services for free.^15,16^ Previously, only one self-sustaining intervention for TB has been described; this had been implemented as a pilot.^17^ Experiences of innovative approaches for sustaining TB services conducted at scale can therefore be instructive for implementers and policymakers. The aim of the present study was to evaluate the impact on TB case detection of a social enterprise intervention that was conducted at scale in Pakistan and to assess its financial sustainability.

## METHODS

### Study design and setting

We used a retrospective, cross-sectional design. While the intervention was conducted in 19 districts of Pakistan, this study describes data from Karachi, Pakistan’s largest city, where the SEM intervention was concentrated. The intervention was first piloted in Karachi in 2015, and subsequently scaled up in 2017. With a population of 13.9 million people in 2014, the number of incident TB cases in Karachi was estimated at 37,000 per year.^2,18^ Prior to the intervention in 2014, 18,104 TB cases (less than half of the estimated total) were notified. Among notified cases, 12,422 (69%) were reported from secondary and tertiary care hospitals, 2,987 (16%) were from the public sector and charity-run primary care facilities, whereas 2,696 (15%) were reported from existing PPM interventions. A low proportion of notifications from private primary providers, despite their large share in the healthcare sector, suggested that they were insufficiently targeted.

### Intervention design

#### Overview of centres

The key feature of the SEM intervention were purpose-built diagnostic and treatment facilities, branded as *Sehmatmand Zindagi (SZ) Centers.* The centres were setup and operated by Community Health Solutions (CHS; Karachi, Pakistan), a private, non-profit enterprise established by individuals with a background in medicine, public health and social entrepreneurship. The centres received start-up funding from the Stop TB Partnership, and were later scaled up through support from the Global Fund. A key feature in the design and operations of the centres was leadership by field staff with experience of working in TB and community-based healthcare. The centres provided coverage in most locations of the city, except for high-income neighbourhoods, corporate zones and military cantonments. The centres consisted of a digital X-ray, a biosafety level-2 (BSL-2) laboratory for processing of sputum samples, a reception, a medical officer’s chamber, a phlebotomy counter and a waiting area. Staffing consisted of a medical officer, a medical receptionist, a laboratory technician and a clinical nurse. The centres were designed with airborne infection control principles with large open fronts and fans for ventilation. Basic furnishing and fixtures were utilised to target a low-to-middle income market. Data were recorded in a custom-developed Health Management Information System (HMIS), and stored on a cloud server.

### Value proposition and revenue model

**Figure 1.**
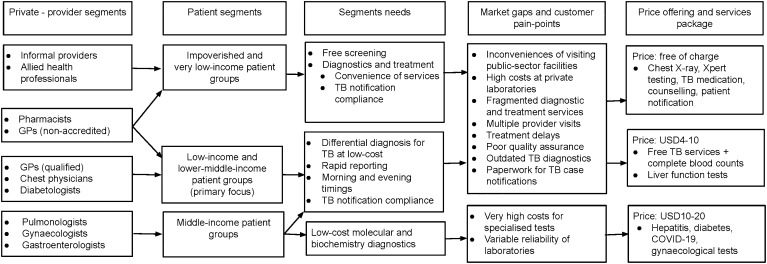
Value proposition and service offerings of TB social enterprise centres to patients and providers in Karachi, Pakistan (2014–2020). USD = US dollar.

A person with respiratory ailments faces several challenges in receiving care within Karachi’s fragmented health system (Figure 1). While public sector hospitals offer services free of cost, they are often located at significant distances with long waiting times. Primary care centres often lack equipment (such as X-rays) and trained staff for diagnosing TB. Public sector services are available only in the daytime, which increased the opportunity cost for daily-wage earners. While offering more convenient timings, private facilities are expensive and quality of care is variable. People often required multiple visits between clinics, laboratories and pharmacies, leading to delays in treatment. Laboratories utilise outdated diagnostics for TB, leading to under-diagnosis, as well as high rates of empirical treatment.^8^

The value proposition of the SZ Centers was integration of TB services, including screening, diagnosis and treatment, along with routine diagnostics, in a single facility with extended timings and multiple locations throughout the city. Quality of care was improved through better diagnostics, including digital X-rays and Xpert^®^ MTB/RIF (Cepheid, Sunnyvale, CA, USA) testing. Rapid turnaround around time for testing, onsite physical examination by physicians and counselling by community workers for treatment helped reduce waiting times and delays. It was anticipated that better geographic access, convenient timings and improved service delivery would increase TB case detection by engaging underserved or non-participating population segments.

The revenue model was of cross-subsidisation, whereby TB services were offered free-of-cost and fees from other diagnostic tests were utilised to generate revenue. The two service lines complemented each other: high-quality TB services helped increase visitor flow to the centres where testing other than TB could be commercially offered, whereas the availability of routine laboratory tests at the same visit increased convenience for people with presumptive TB. These services were often promoted as bundled packages, increasing overall value.

The average cost involved in diagnosing and notifying a case has been described as ranging from USD45–13,417, depending on the setting and case detection strategy.^9,19^ A recent analysis from Pakistan determined an average cost of USD223 per case diagnosed, of which personnel (26%), incentives (18%) testing (6%) and TB medication (17%) were the major cost items.^20^

### Private sector engagement and marketing

Providers were engaged through a strategy called direct-to-physician marketing. Each SZ Center was supported by a medical representative (MR), who surveyed and mapped all private healthcare facilities in the SZ Center’s catchment area and engaged providers not already notifying to the NTP. This prevented overlap with existing PPM interventions. The MR primarily focused on small-scale providers and the informal sector. Existing PPM models relied on a large field workforce with multiple responsibilities, including engaging providers, transporting samples, delivering medication and completing paperwork. In the SEM approach, the primary responsibility of an MR was to generate referrals to the SZ Center. Basic training on TB awareness, symptomatology and referrals were provided through onsite detailing on tablet devices and small group training sessions. A shorter and simpler engagement process allowed each MR to target a wider network of providers, relative to other PPM models. Referral prescription pads that promoted the centres’ service packages were provided. Providers were supported with various monetary incentives to encourage referrals for laboratory diagnostic tests, ranging from 5% to 25% of the total value of the test. As per NTP policy, all PPM implementers offered USD3.50 per case diagnosed to private-providers. Below-the-line (BTL) marketing involved the use of posters and charts outlining various services of the centres, and were placed in waiting areas of partnering clinics and pharmacies. Prominent backlit boards were placed at the centres, banners were placed in the vicinity and pamphlets were distributed to encourage walk-in traffic. A third strategy involved active case-finding (ACF) using mobile X-ray units in the community. Individuals from all three strategies were screened, tested, treated and notified in a standardised approach under existing NTP guidelines once they reached the centres.

### Statistical analysis

Programmatic data from the Karachi centres from 2017 to 2020 were utilised, when the intervention had been implemented at scale. To determine yields from the private sector intervention alone, cases detected from the ACF intervention were excluded. Aggregated data reports for CXR, Xpert tests, and the number of cases detected and enrolled for treatment were extracted from the HMIS and analysed for frequency statistics. Actively engaged referral providers were defined as any private sector provider who had sent at least one patient to an SZ Center in Karachi in the calendar year. Notifications reported to the NTP from all of Karachi’s facilities and interventions were evaluated from 2014 to 2020. Absolute increase in TB case detection in Karachi was calculated using two baseline years, 2014 and 2016, when the intervention was piloted and scaled-up, respectively. All CHS notifications were included for the additionality analysis, including those through ACF for the additionality calculations.

### Financial analysis

Total revenue (TR) from actual centre sales was obtained. Gross profit for each test was calculated by subtracting price from directs costs that consisted of testing, calibration reagents and consumables. As the profit margins differed between tests, their contribution mix (proportion of each test among all tests) was first calculated and multiplied with their gross profits to calculate the weighted average gross profit. The weighted averages were summed to calculate the direct costs from each unit of sale (determined to be 23%). Annual gross profit (GP) was calculated by subtracting total revenue from cost of diagnostic sales. Annual centre costs were determined utilising expenses recorded for the centres. Major cost items included staffing, rent, utilities, security and maintenance. Costs of Xpert cartridges were separately added (USD9.98 per test). Annual net operating loss was determined by subtracting annual operating costs from annual gross profit. The operating loss was divided by the number of drug-susceptible (DS-TB) and drug-resistant TB (DR-TB) and cases notified to determine the donor cost per TB case notified. Operating cost share was defined as the proportion of operating costs covered through commercial sales.

### Ethical approval

As the study only utilised aggregate programmatic, and no patient-level data, it was not considered eligible for ethical review.

## RESULTS

Total providers linked to the centres increased from 987 in 2017 to 2,788 in 2019 (Table 1). During 2017–2020, 171,488 people underwent screening for TB, among whom 107,341 (62.6%) were tested using Xpert, and 10,570 (9.8%) had bacteriologically positive TB (MTB+). A total of 9,302 individuals with MTB+ (88.0%) were enrolled for treatment and notified. A further 7,142 (38.2%) were empirically treated, and 2,239 individuals (12.0% of all notified) were enrolled for extrapulmonary TB treatment. An additional 197 individuals (1.1% of MTB+) were diagnosed with DR-TB. In total, the centres notified and treated 18,683 individuals over 4 years at a treatment success rate of 85.2%. The loss to follow-up rate was 8.2%, and 3.3% individuals died during treatment. The SZ Centers’ annual TB notifications increased from 3,111 in 2017 to a maximum of 5,420 in 2019 and decreased to 5,348 in 2020.

**Table 1.** Screening, diagnosis and treatment indicators for TB social enterprise centres in Karachi, Pakistan (2017–2020).

	2017	2018	2019	2020	Total (2017–2020)	Mean/(% of total)
	*n*	*n*	*n*	*n*	*n*	
Private sector network						
Diagnostic centres	31	31	24	24	24	—
Engaged referral providers	987	2,271	2,788	2,165	2,788	—
Screening and diagnostics						
Chest X-rays	26,647	47,294	51,635	45,912	171,488	42,872
Xpert MTB/RIF tests	20,704	29,178	31,478	25,981	107,341	62.6%
MTB+ detected	1,874	2,829	2,894	2,973	10,570	9.8%
Notifications						
Total TB notifications	3,111	4,804	5,420	5,348	18,683	4,671
Bacteriologically positive TB	1,331	2,441	2,732	2,798	9,302	49.8%
Clinical (empirically treated) TB	1,621	1,893	1,943	1,685	7,142	38.2%
Extrapulmonary TB	159	470	745	865	2,239	12.0%
Multidrug-resistant TB	11	70	66	50	197	1.1%
Outcomes, %						
Treatment success	84.0	79.8	88.6	88.4	85.2	—
Lost to follow-up	12.6	12.7	4.2	3.1	8.2	—
Died during treatment	1.6	3.2	4.0	4.3	3.3	—
Transferred to DR-TB sites	0.4	1.5	1.2	0.9	1.0	—
Transferred to other DS-TB sites	1.4	2.8	2.0	3.2	2.3	—
Chest X-ray referrals and TB notification ratios						
Chest X-rays per provider	27.0	20.8	18.5	21.2	—	21.9
TB notifications per provider	3.2	2.1	1.9	2.5	—	2.4
Chest X-rays per centre	860	1,526	2,151	1,913	—	1,612
TB notifications per centre	100	155	226	223	—	176

MTB+ = M. tuberculosis-positive; DS-TB = drug-susceptible TB; DR-TB = drug-resistant TB.

**Figure 2.**
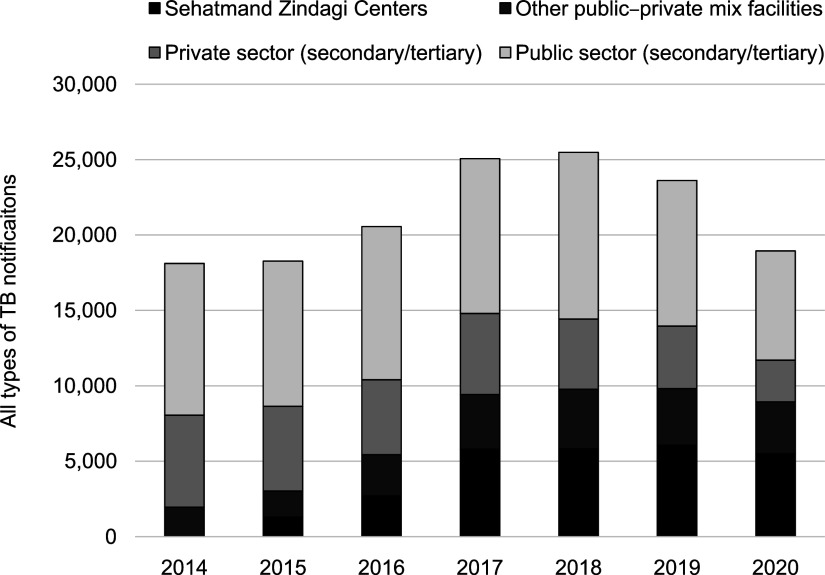
Annual drug-susceptible TB notifications in Karachi, Pakistan (2014–2020).

Annual total TB notifications in Karachi increased from 18,105 cases reported in 2014, to a maximum of 25,840 cases in 2018 (Figure 2). This represents a 40% increase over the baseline year of 2014, and an increase of 26% from 2016, prior to the intervention scale-up. The proportion of CHS notifications among all cases reported in Karachi increased from 7% in 2015 to 23% in 2018, and reached 29% in 2020 during the COVID-19 pandemic. Notifications from other PPM interventions also increased during this period (increase of 92% from 2014). Notifications from public- and private-sector facilities did not vary significantly, before declining in 2020.

Commercial revenue from laboratory testing increased from USD89,762 in 2017 to USD288,065 in 2020, and gross profits increased from USD49,369 in 2017 to USD158,436 in 2020 (Table 2). The centres’ operating loss decreased from USD937,993 in 2017 to USD890,405 in 2020. The average cost per case notified during this period was USD203, decreasing from USD302 in 2017 to USD166 in 2020. Average cost per DR-TB case notified was USD19,214. Operating cost share supported through commercial diagnostic sales increased from 5% in 2017 to 15% in 2020.

**Table 2.** Summary of financial indicators for TB social enterprise centres in Karachi, Pakistan (2017–2020).

	2017	2018	2019	2020	Total
	USD	USD	USD	USD	USD
Commercial revenue	89,762	175,954	238,967	288,065	792,747
Cost of diagnostic sales	–40,393	–79,179	–107,535	–129,629	–356,736
Gross profit	49,369	96,775	131,432	158,436	436,011
Centre costs	–780,736	–848,626	–731,065	–789,550	–3,149,977
Xpert testing costs	–206,626	–291,196	–314,150	–259,290	–1,071,263
Operating costs	–987,362	–1,139,822	–1,045,215	–1,048,840	–4,221,240
Net operating loss	–937,993	–1,043,048	–913,784	–890,405	–3,785,229
Donor cost/case notified	302	217	169	166	203
Donor cost/DR-TB notified	85,272	14,901	13,845	17,808	19,214
Donor cost share, %	95	92	87	85	90

## DISCUSSION

Prior to the SEM intervention, TB notifications in Karachi were concentrated among large public- and private-sector hospitals while smaller scale, less-qualified primary providers were being missed. A combination of convenient locations, extended timings and one-window TB services helped create a unique value proposition for lower- and middle-income segments, who were reluctant to visit existing TB facilities. This was supported by localised, BTL marketing and promotion of referrals of people with presumptive TB at private clinics that were not already linked to the NTP. Such an approach is often referred to as the ‘blue ocean’ marketing strategy, whereby new, uncontested markets are created through innovation in either the product design or the channels with which non-users are engaged.^21^ By avoiding direct competition with established TB care facilities and existing PPM interventions, the overall size of the ‘market’ for TB services expanded and additional people were detected (increase of 40% over the baseline).

The COVID-19 pandemic adversely impacted TB notifications in Karachi, and the focus of the centres shifted to ensuring treatment continuation.^22^ Visitor numbers recovered later in 2020, and the centres maintained previous levels of case-detection. Total TB notifications in Karachi, however, decreased by 20%, as notifications from tertiary care hospitals significantly declined. Consequently, the share of the cases notified through the centres increased to nearly one-third.

The intervention’s cost recovery through commercial diagnostic testing was less encouraging due to high indirect (overhead) costs. Our findings suggest that in order to achieve financial sustainability, organisations must be highly cost-conscious, in addition to pursuing revenue growth. However, with a high proportion of people seeking care in the private sector, resources from the government can be used to expand the reach of the TB programme at lower costs, relative to setting up these services in their entirety in the public sector.

A qualitative assessment of the benefits and limitations of the SEM approach relative to conventional models has been described, based on our experience of having implemented various PPM strategies (Table 3). Policymakers and implementers are encouraged to review these considerations when planning SEM interventions, keeping in view the health systems structure and TB epidemiology in their countries. The pilot intervention in Dhaka, Bangladesh, where three, similar one-window TB centres were set up, diagnosed 10,288 TB cases from 2014 to 2017, and revenue generation grew to USD146,645 per year.^17^ A third SEM intervention for TB has recently been set up in Manila, The Philippines.^23^

**Table 3.** Limitations and benefits of the social enterprise model compared to conventional public-private mix (PPM) approaches.

Considerations	Limitations	Benefits
Financial	Requires upfront capital investment to set up centres	Capital investments may eventually yield positive financial return to funders and investors
	Less cost-effective in the short term as business matures and market is created	May yield higher cost-effectiveness over the medium term with increasing profits and case-notifications
	Working capital required to operate centres over the medium term	Social impact can be driven for multiple public health diseases using the same infrastructure
Case-Detection	Potentially lower yields per private provider relative to conventional PPM approaches	Wider engagement with private sector, including informal practitioners and pharmacies
	Healthcare expenses incurred by people with presumptive TB	User fees significantly lower than those offered by competing laboratories in the private sector
	May lead to duplication of services with other public and private sector facilities	Centre locations may be prioritised to focus on underserved areas, and self-referrals of people with TB symptoms can also be promoted
	Less useful in countries with gaps in private-sector notifications only and not in case detection	Model can be adapted for other TB elimination strategies such as TB preventive or drug-resistant treatment
Managerial	Involves greater challenges in staff recruitment, retention and training	Allows local capacity-building for responding to TB and other infectious diseases
	Conflicting job-descriptions with financial and social ‘double bottom-lines’, i.e., staff performance evaluated on both cases diagnosed and revenue	Staff from diverse sectors support a creative culture, leading to greater innovation and efficiency
	Separation of cost-accounting, financial budgeting and reporting systems for donor-funded and commercial operations	Strong financial and monitoring systems can help reduce costs and attract private-capital to augment donor-funded investments
Technical	Requires resources for operating, maintaining and repairing medical equipment	Improved sensitivity of screening and bacteriological positivity relative to conventional approaches using sputum smear microscopy and verbal screening
	Recording and reporting of diagnostics requires supportive health information infrastructure	Digital systems can improve programme efficiency, targeting of interventions and treatment outcomes

PPM = public-private mix.

This analysis was limited by assigning total costs to both DS- and DR-TB interventions to determine cost per case detected. An activity-based costing approach was beyond the scope of this analysis. This would lower the cost per case by spreading operating costs across both interventions. Costs of equipment purchase were excluded from the analysis.

## CONCLUSION

An innovative approach of private-sector engagement using a social enterprise model increased TB case detection in Karachi. The model achieved modest operating cost recovery by generating revenue through commercial laboratory diagnostics.
